# Detecting Selective Protein Binding Inside Plasmonic Nanopores: Toward a Mimic of the Nuclear Pore Complex

**DOI:** 10.3389/fchem.2018.00637

**Published:** 2018-12-21

**Authors:** Bita Malekian, Rafael L. Schoch, Timothy Robson, Gustav Ferrand -Drake del Castillo, Kunli Xiong, Gustav Emilsson, Larisa E. Kapinos, Roderick Y. H. Lim, Andreas Dahlin

**Affiliations:** ^1^Department of Chemistry and Chemical Engineering, Chalmers University of Technology, Gothenburg, Sweden; ^2^Biozentrum and the Swiss Nanoscience Institute, University of Basel, Basel, Switzerland

**Keywords:** plasmons, nanopores, nuclear pore complex, proteins, sensors

## Abstract

Biosensors based on plasmonic nanostructures offer label-free and real-time monitoring of biomolecular interactions. However, so do many other surface sensitive techniques with equal or better resolution in terms of surface coverage. Yet, plasmonic nanostructures offer unique possibilities to study effects associated with nanoscale geometry. In this work we use plasmonic nanopores with double gold films and detect binding of proteins inside them. By thiol and trietoxysilane chemistry, receptors are selectively positioned on the silicon nitride interior walls. Larger (~150 nm) nanopores are used detect binding of averaged sized proteins (~60 kg/mol) with high signal to noise (>100). Further, we fabricate pores that approach the size of the nuclear pore complex (diameter down to 50 nm) and graft disordered phenylalanine-glycine nucleoporin domains to the walls, followed by titration of karyopherinβ1 transport receptors. The interactions are shown to occur with similar affinity as determined by conventional surface plasmon resonance on planar surfaces. Our work illustrates another unique application of plasmonic nanostructures, namely the possibility to mimic the geometry of a biological nanomachine with integrated optical sensing capabilities.

## Introduction

Biosensors can be divided into two categories: devices aiming to detect analytes from complex samples or tools used to characterize biomolecular interactions in a controlled environment. The first category is certainly associated with important applications but real devices often suffer from problems with specificity, i.e., other molecular species than the analyte often interfere and cause false positives (Zhang et al., 2017). The second category is especially suitable for generic techniques that detect essentially anything binding to a surface. If a receptor is immobilized on the sensor surface and the target added in a pure buffer, the risk of interfering events is much smaller. Furthermore, using surface sensitive techniques, biomolecular interactions can be studied label-free and in real-time. The golden standard for this type of biosensors has since long been optical detection by surface plasmon resonance (SPR) (Homola, 2008), which responds to changes in refractive index within a few hundred nm of a metal surface. More recently, so called nanoplasmonic sensors have emerged (Jackman et al., 2017), which operate in a similar manner by utilizing nanostructured metals. The plasmons excited in such structures are typically associated with a shorter probing depth (tens of nm) and spectroscopy is normally performed in transmission mode or dark field illumination (Lopez et al., 2017).

However, nanoplasmonic sensors are also associated with several disadvantages. First, quantification of the bound amount is difficult compared to SPR (Emilsson et al., 2015), for which the planar surface only allows variation in one spatial dimension. Second, surface functionalization is more complex as several materials tend to be exposed in the nanostructures (Oliverio et al., 2017), most often gold and silica (Marie et al., 2007), again in contrast to SPR. In addition, the resolution in terms of surface coverage, which arguably is the most relevant parameter in most situations (Dahlin, 2012), is typically similar to SPR at best. This suggests limited use of nanostructured plasmonic sensors for studying biomolecular interactions, at least through ordinary refractometric detection. Still, if one can utilize the structure itself to address questions related to nanoscale geometry, the sensors offer unique opportunities not possible to achieve by techniques based on planar surfaces. For instance, we have used various types of plasmonic nanopores to investigate biomolecular interactions on curved lipid membranes (Junesch et al., 2015) and the ability of polymer brushes to form barriers toward proteins (Emilsson et al., 2018). The different materials exposed may also turn into an advantage because appropriate chemistry makes it possible to direct molecular binding selectively to certain regions of the nanostructure, thereby reducing the active area (Spackova et al., 2018). This may increase the signal in systems where mass transport limitations and irreversible binding prevents equilibrium establishment between the receptors and a fixed uniform analyte concentration (Feuz et al., 2010, 2012). To date, relatively few studies have been presented where receptors are selectively positioned inside nanopores (Emilsson et al., 2018), although it has been achieved for nanoholes (Marie et al., 2007; Ferreira et al., 2009; Feuz et al., 2010) (having a bottom surface).

In this work we use plasmonic nanopores with two gold films (Dahlin et al., 2014) to detect protein binding to the interior silicon nitride walls. We present protocols for specific modification of gold and silica such that receptors can be immobilized solely inside the pores by covalent bonds. The plasmonic response upon protein binding and the performance of the sensor is evaluated, especially with respect to pore diameter. Finally, a mimic of the nuclear pore complex (Jovanovic-Talisman and Zilman, 2017) (NPC) is presented by end-grafting phenylalanine-glycine (FG) rich nucleoporin domains inside the pores and detecting concentration dependent binding of the nuclear import receptor karyopherinβ1 (Kapβ1) (Kapinos et al., 2017). This illustrates the possibility of understanding biological “nanomachines” by a bottom up approach based on engineered nanostructures where the plasmonic activity serves as a useful tool to obtain information in a label-free manner.

## Experimental

### Chemicals

All chemicals were from Sigma unless specified otherwise. Polystyrene-sulfate colloids were from Microparticles GmbH. Thiolated PEG was from Laysan Bio. The yeast Nsp1p FG-fragment Nsp1p-12FF (residues 262–492; 1xFG, 11xFSFG) was cloned and expressed as described previously (Wagner et al., 2015). The fragment contains two N-terminal cysteine residues for chemical crosslinking. Protein binding experiments were performed in phosphate buffered saline (PBS).

### SPR

SPR measurements were performed on a SPR NaviTM 220A instrument (BioNavis) equipped with three laser wavelengths: 670, 785, and 980 nm. SPR sensor surfaces with a 50 nm thick gold layer were used. A 50 nm thick SiO_2_ layer was deposited by chemical vapor deposition on the gold surface (Surface Technology Systems). Dry scans of the sensor surface were measured prior to and post-deposition of SiO_2_ and silanization with APTES.

### QCMD

Measurements were performed on a Qsense E4 instument (Biolin Scientific) with plain gold or silica coated crystals. Data is shown for the 5th overtone and normalized.

### Nanofabrication

The fabrications process has been described previously (Dahlin et al., 2014). For all samples colloids ~150 nm in diameter were used but the batches were not exactly the same, which gives some variation in characteristic spacing (Dahlin et al., 2014). There was also some variation in silicon nitride membrane thickness (±20 nm) between different samples. Prior to experiments samples were cleaned with “basic piranha” (1:1:5 by volume of conc. NH_3_, H_2_O_2_, and water) at 80°C for 10 min, washed with ethanol and/or treated with low power O_2_ plasma.

### PEG-Modification of Au

Samples were incubated in 0.5 mM 2 kg/mol PEG or 0.1 mM 5 kg/mol PEG (Laysan Bio) in PBS overnight. For these relatively short PEGs a brush with good anti-fouling properties can be formed without using cloud point grafting (Emilsson et al., 2015).

### Silanization

For solvent phase silanization, samples were exposed to 1% APTES in methanol or toluene for 30 min. For vapor phase silanization, a drop of APTES was placed on a glass slide next to the sample in a chamber connected to a pump. Pressure was lowered to ~2 mbar after which the vacuum chamber was sealed. After 30 min the pressure was increased to ambient conditions and samples were annealed at 100°C for 30 min. Following annealing the samples were rinsed in ethanol and milli-Q water and dried in nitrogen.

### Biotinylation

25 mM EDC (1-ethyl-3-(3-dimethylaminopropyl)carbodiimide hydrochloride) and 8.3 mM sulfo-NHS (N-hydroxysulfosuccinimide) were introduced together with 2 mM biotin-EG_11_-COOH in 20 mM MES buffer pH 5.5.

### FG-Domain Grafting

For activation of the APTES amine groups, 2 mg of sulfosuccinimidyl 4-(N-maleimidomethyl) cyclohexane-1-carboxylate (sulfo-SMCC, ThermoFisher Scientific) was dissolved in 1 mL warm H_2_O, mixed with 2 mL of PBS and introduced to the sample in the flow cell for 30 min. All solutions used in the flow cells were degassed using a vacuum pump while stirring with a magnetic stirrer. Binding of cNsp1 was done in the presence of 2 mM tris(2-carboxyethyl) phosphine hydrochloride in PBS to prevent disulfide formation. The cells were then rinsed with PBS and 10 μM of HS-(CH_2_)_11_-(OCH_2_CH_2_)_3_-OH (Nanoscience, USA) in PBS for ~10 min to quench remaining active APTES sites.

### Imaging Kapβ1 Colloids

The coating of 10 nm diameter gold nanoparticles followed a procedure relying on straightforward electrostatic adsorption of proteins to the negatively charged gold particles (Hughes, 2005). Briefly, 7.5 mL of the nanoparticle suspension (OD 1) in 0.1 M PBS was mixed with 250 μL of a 1% PEG 3350 Da solution in H_2_O and adjusted to pH 5.2 (i.e., 0.5 points above the pI of Kapβ1). Subsequently the colloids were added to Kapβ1 to obtain a 200 nM Kapβ1 suspension and stabilized after 2 min by adding 1 mL of 1% PEG in H_2_O. The cNsp1 functionalized nanopore samples were then incubated with the colloidal suspension in PDMS flow cells for 1 h and proteins were crosslinked using 200 mM EDC, 400 mM NHS in 20 mM MES buffer pH 6.0, 100 mM NaCl, for 1 h. The sample was rinsed with PBS, H_2_O, dried using a N_2_ gas stream and mounted for scanning electron microscopy imaging.

## Results and Discussion

Plasmonic nanopores consisting of two 30 nm thin gold films on both sides of a ~50 nm silicon nitride membrane (100 × 100 μm^2^) were prepared as described previously (Dahlin et al., 2014). In brief, colloidal self-assembly is used to create short-range ordered pattern of pores which enables excitation of propagating surface plasmon modes. The pore diameter was varied by oxygen plasma treatment of the colloids before metal deposition (Xiong et al., 2016). The plasmonic activity was confirmed by measuring the extinction spectra on the microscale as described previously (Dahlin et al., 2009). The ultrathin membrane results in coupling between the metal films and slightly blue-shifted resonances (compared to pores in a single gold film) but no Fabry-Perot type interference in the visible region (Dahlin et al., 2014). We noted that especially for smaller pores it became increasingly difficult to remove the colloids from the membrane without breaking it. One way to circumvent this problem is to simply have many membranes on the same chip (we used a size of 18 × 24 mm^2^) and measure on those that remain intact through the whole fabrication process. Since all steps are parallel rather than serial, including the colloidal lithography step, having multiple membranes does not lead to an increase in time and cost of fabrication (per chip). Some pore arrays were prepared by electron beam lithography (unpublished results) and those samples behaved the same way in the sensing experiments as the samples prepared by colloidal lithography.

**Figure 1:**
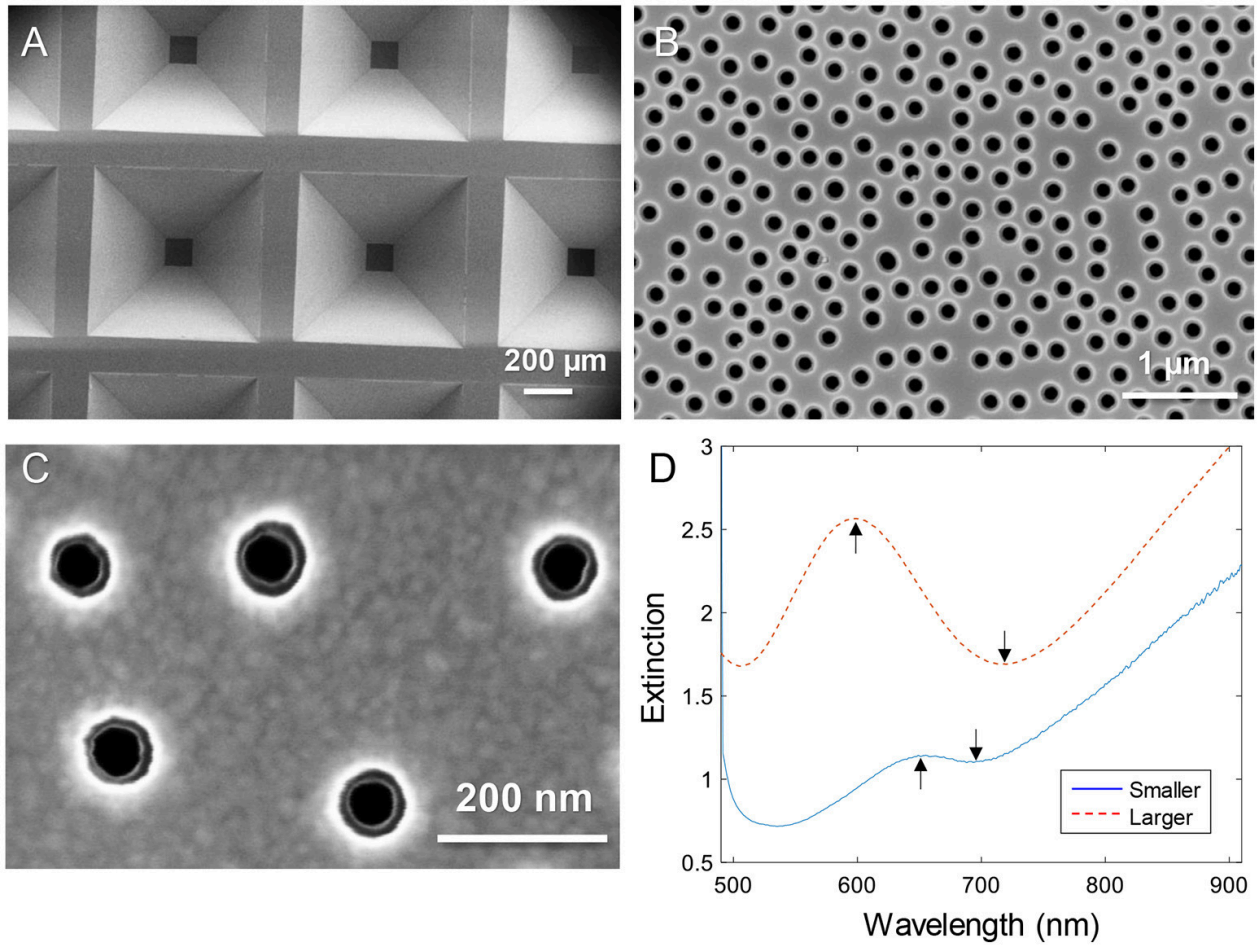
Nanopores in silicon nitride membranes with two thin gold films. **(A)** Arrays of membranes supported by the wafer imaged from the backside. **(B)** Example of “larger” nanopores imaged from the topside. **(C)** Close up showing “smaller” nanopores imaged from the topside. Note that the backside diameter is also visible. **(D)** Typical extinction spectra in a water environment for larger and smaller diameters. The arrows show the spectral resonance features (peak and dip).

Figure 1 shows electron microscopy images of nanopore samples. The silicon nitride membranes supported by the silicon wafer (Dahlin et al., 2014) are shown in Figure 1A. The nanopores were found to have slightly inclined walls as expected since the dry etching is not entirely isotropic (Junesch et al., 2012; Dahlin et al., 2014). By imaging from the top side, both the upper and lower diameters become visible (with the right brightness/contrast settings). Figure 1B shows an example of pores with upper diameter 170–180 nm and lower diameter 110–120 nm. Figure 1C shows a close up of a sample with pores that have an upper diameter of 80–90 nm and a lower diameter of 50–60 nm. Throughout this work these were the two sizes of pores used and they are simply referred to as “larger” and “smaller.” The variation in diameter of ~10 nm observed when measuring many pores is expected and originates from the size distribution of the polystyrene-sulfate colloids used for the lithography (Xiong et al., 2016).

The extinction spectra of larger and smaller pores in a water environment are shown in Figure 1D. The extinction peak was mainly sensitive to the characteristic spacing between the colloids and the thickness of the membrane sandwiched between the gold films (Dahlin et al., 2014). In analogy with nanoholes in single thin gold films (Xiong et al., 2016), reducing the diameter lead to a strong reduction in resonance strength (extinction peak magnitude) and the dip was blue shifted closer to the peak wavelength (Figure 1D). The smaller pores are clearly just at the limit for when the resonance can be identified in the spectrum. It should be noted that the plasmonic response is highest at the pores due to the near field distribution (Dahlin et al., 2014). Especially for the dip resonance of smaller pores, the sensitivity is expected to be focused entirely to the pore interior (Xiong et al., 2016).

**Figure 2:**
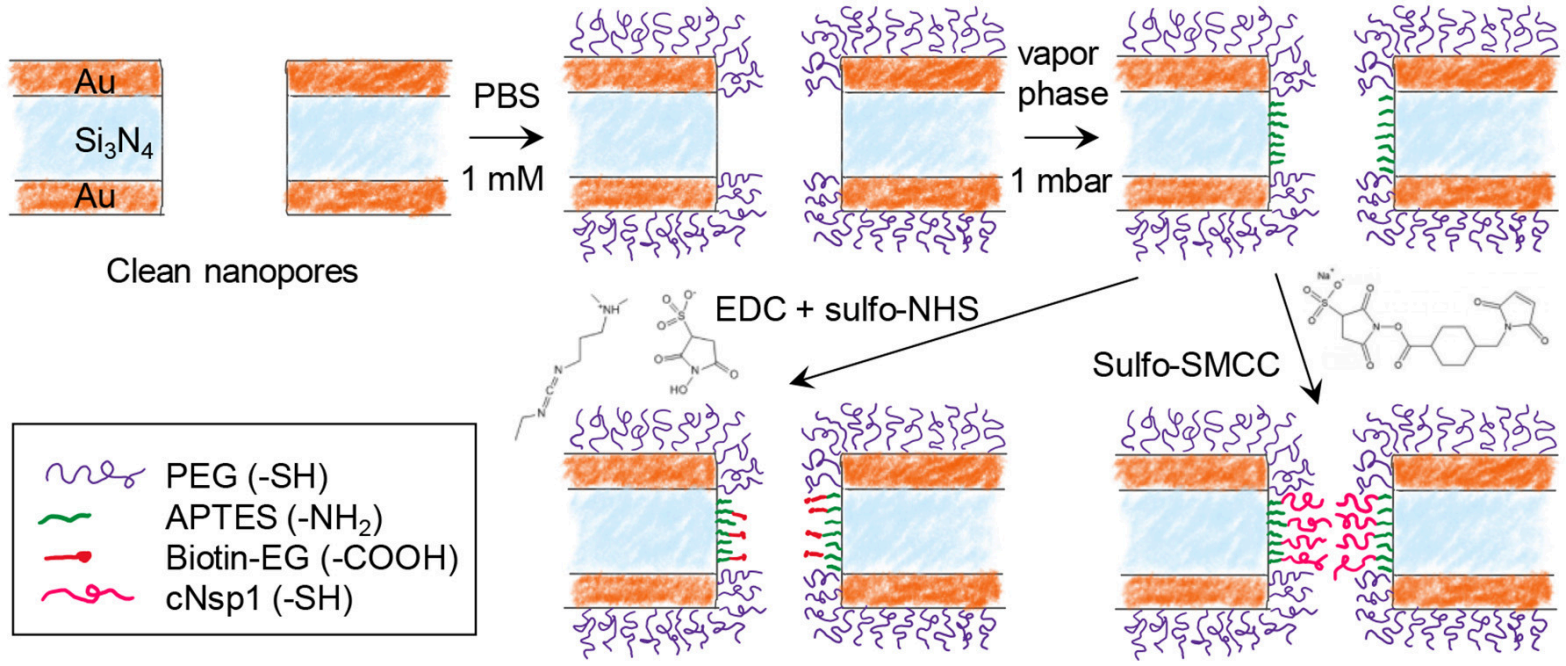
Chemical functionalization strategy for immobilizing receptors inside nanopores. After modifying gold with PEG, the nanopore walls are modified with APTES. Receptors can then be bound by cross-linking chemistry between -NH_2_ and -COOH (EDC + sulfo-NHS) or between -NH_2_ and -SH (sulfo-SMCC). Using sulfo-SMCC is more challenging as it requires that the thiol-PEG coating prevents other thiol groups from reaching the gold.

Figure 2 shows the steps for chemical functionalization of the nanopores for selective protein binding to their interior. All steps were verified independently on planar gold and silica using SPR and quartz crystal microbalance with dissipation monitoring (QCMD). The first step in the surface functionalization process was always to selectively render gold inert by binding relatively short chains (2 or 5 kg/mol) of thiolated poly(ethylene glycol) (PEG). As we have shown previously, this makes gold highly inert toward protein absorption (Emilsson et al., 2015) and leaves silica available for subsequent functionalization steps (Xiong et al., 2016). At the same time, the PEGs are not long enough to seal the apertures (Emilsson et al., 2018).

The next step was silanization of the silicon nitride walls using (3-aminopropyl)triethoxysilane (APTES). We assumed that a native oxide layer existed on the nitride, especially due to the oxidative cleaning protocols before experiments. Thus, the nanopore walls should behave essentially as a silica surface with respect to chemical modifications. APTES binding was tested in both vapor (2 mbar, room temperature) and solvent phase (toluene or methanol). Based on results from planar surfaces analyzed in SPR (silica coated gold sensor slides), vapor phase was considered a better method as it provided results similar to what is expected from a complete APTES monolayer (see below). After solvent phase silanization, thicker layers were obtained (several nm), indicating some formation of multilayers and inhomogenous films as expected (Zhu et al., 2012). Nevertheless, even with solvent phase silanization, APTES functionalization was successful qualitatively.

In order to investigate the response from a model protein we functionalized the nanopore walls with biotin after APTES. This was achieved by EDC/NHS conjugation between the amine on APTES and the carboxylic acid on the modified biotin (Figure 2). In order to create an NPC mimic, FG-domains were grafted to the walls by instead using activation with sulfo-SMCC, which cross links thiol with amines. We used a cysteine-modified FG-domain construct (Schoch et al., 2012; Wagner et al., 2015), i.e., the FG-rich domain from the yeast nucleoporin Nsp1 (referred to as cNsp1) due to its low propensity for aggregation.

**Figure 3:**
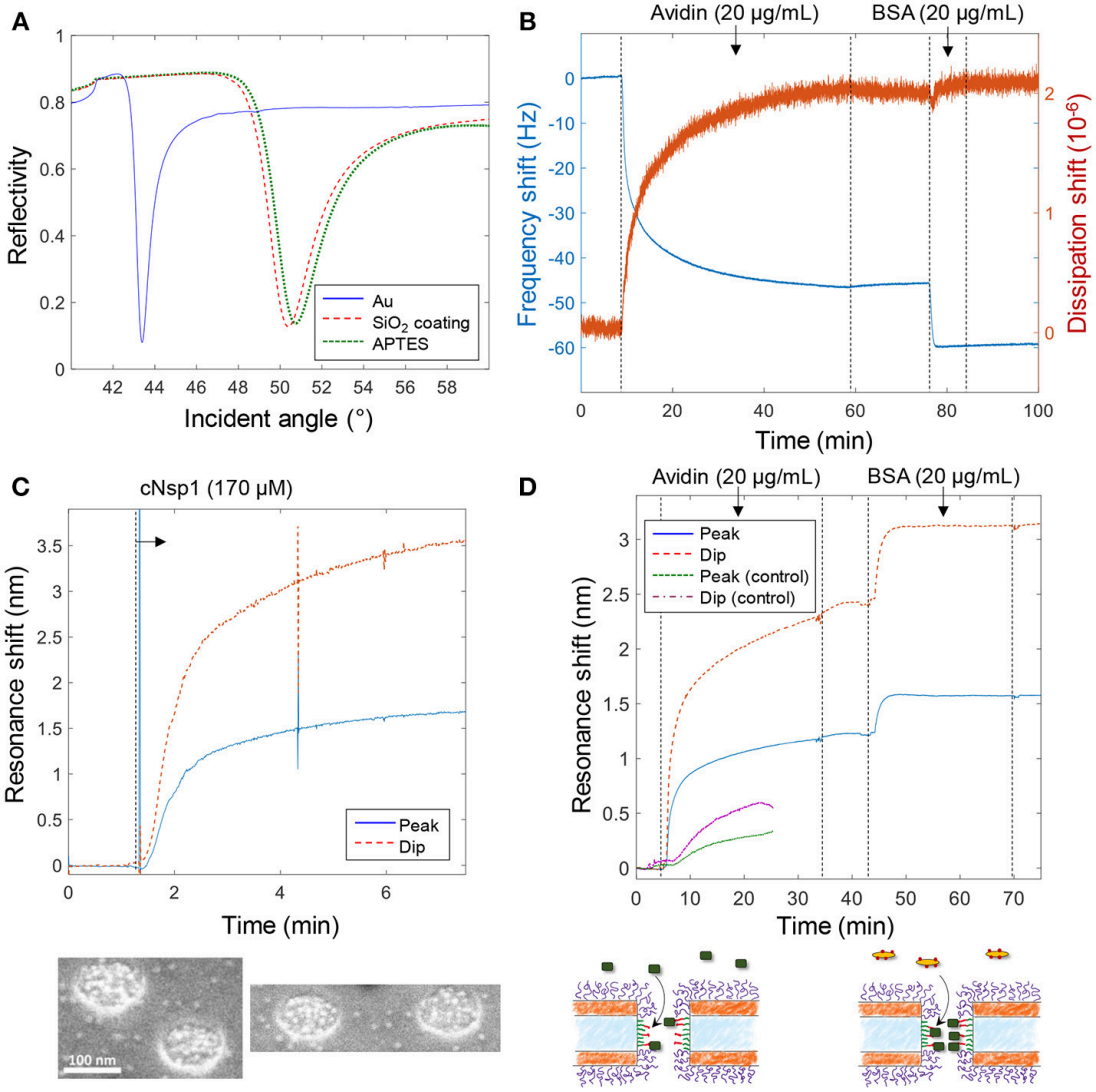
Verification of surface functionalization and model protein binding. **(A)** Angular SPR reflectivity spectra in air (wavelength 670 nm) confirming APTES binding to silica. **(B)** QCMD data showing avidin and subsequent biotin-BSA binding to silica sensor crystal modified with APTES and biotin (as in Figure 2). **(C)** Plasmonic signal from nanopores showing grafting of cNsp1 in PBS buffer to larger pores (after activation by sulfo-SMCC). The images below show enrichment of Kapβ1-modified gold colloids inside pores. **(D)** Detection of avidin and biotin-BSA binding inside larger nanopores, also including a control where no biotinylation was performed on the APTES layer (which gives less and slower binding). The schematics below illustrate the binding events.

Figure 3 shows various verifications of the surface functionalization steps. Figure 3A shows SPR spectra in air of Au surfaces upon deposition of 50 nm SiO_2_ by chemical vapor deposition. After APTES modification a small but clear shift was measured and the APTES film was estimated to be 1.5 ± 0.3 nm, with refractive index 1.465 (Howarter and Youngblood, 2006), by Fresnel models (Junesch et al., 2012), which we consider to be in good agreement with a complete homogenous monolayer. Figure 3B shows the QCMD response upon binding of avidin and biotinylated bovine serum albumin (BSA) to silica sensor crystals. (Subsequent binding of biotin-BSA is possible since avidin has four binding sites for biotin). No binding of biotin-BSA was observed to APTES modified silica unless avidin was first bound, showing that it is truly the biotin-avidin interaction which results in binding of this second layer.

Figure 3C shows the plasmonic response from the nanopores upon grafting of cNsp1 to the interior walls. As in previous studies, we present both the shift in resonance peak and dip as their relative signal strength contains information about where in the nanostructure molecular binding occurs (Junesch et al., 2015). The ratio between the dip shift and the peak shift is typically around 2 but increases slightly for binding inside nanopores (and decreases for binding outside). However, the contrast in the signals also depends on other factors such as nanostructure dimensions and we have never investigated the expected dip/peak shift ratio for the nanopores with two gold films. Therefore, we used independent tests in SPR and QCMD to verify the surface chemistry (not the ratio between the dip and peak). Additionally, we used scanning electron microscopy to visualize the binding of Kapβ1 coated gold colloids. The inset in Figure 3C shows that the colloids (bright spots) are highly enriched within the nanopores and therefore specifically targeting cNsp1 localized within the pores. Some colloids are visible in between the pores though, which is probably because some cNsp1 becomes chemisorbed to the gold due to its thiol terminal. This illustrates a limitation with the sulfo-SMCC coupling, while the APTES and EDC + sulfo-NHS approach does not have this problem.

Figure 3D shows an example of protein binding to the biotin-functionalized interior walls of nanopores by first introducing avidin and later biotin-BSA. Due to the irreversible interaction, each signal corresponds to a complete monolayer of proteins. Yet we observed that biotin-BSA binding was much faster (at the same concentration) and the signal was consistently lower than for avidin, despite these proteins being similar in molecular weight (~66 kg/mol). The lower signal may to some extent be explained by the plasmonic near field being stronger closer to the surface (Dahlin et al., 2014), which is expected especially for larger pores (Xiong et al., 2016). However, the trend was the same in QCMD (Figure 3B) which has a completely different transduction mechanism. In particular, there was barely any change in dissipation for biotin-BSA binding. We attribute these observations to the high number of biotin molecules conjugated to the BSA. This promotes faster binding and the elongated BSA molecule can likely form a thin compact layer by spreading out, thereby interacting with several avidin molecules. By alternating additions of avidin and biotin-BSA, multilayers of proteins could also be assembled (not shown), in agreement with previous work (Tominaga et al., 2008). As a control experiment we also introduced avidin to nanopores treated identically but without the biotinylation step, which showed some non-specific binding to the APTES-modified silicon nitride walls, but considerably slower and weaker (Figure 3D), confirming that biotin is present inside the pores in the other measurements.

Notably, the signal to noise for protein detection inside the larger pores is fairly high, ~1,000 when considering short term noise over seconds. It was, however, difficult to fully avoid fluctuations upon liquid injection and the occasional diffusion of particles in solution through the detection spot. The measurement spot is ~50 μm in diameter, i.e., ~25% of the membrane area, when in focus (Dahlin et al., 2009). Based on the area inside the pores and the coverage of an avidin monolayer on the order of ~200 ng/cm^2^, there are < 500 proteins inside each pore. Hence the detection limit corresponds to < 1 protein per pore (for a stable baseline). In terms of number of molecules, which is another way to define detection limit instead of analyte concentration in bulk (Ferreira et al., 2009), the signal in Figure 3D originates from ~20 attomoles for a pore density of 10 μm^−2^.

**Figure 4:**
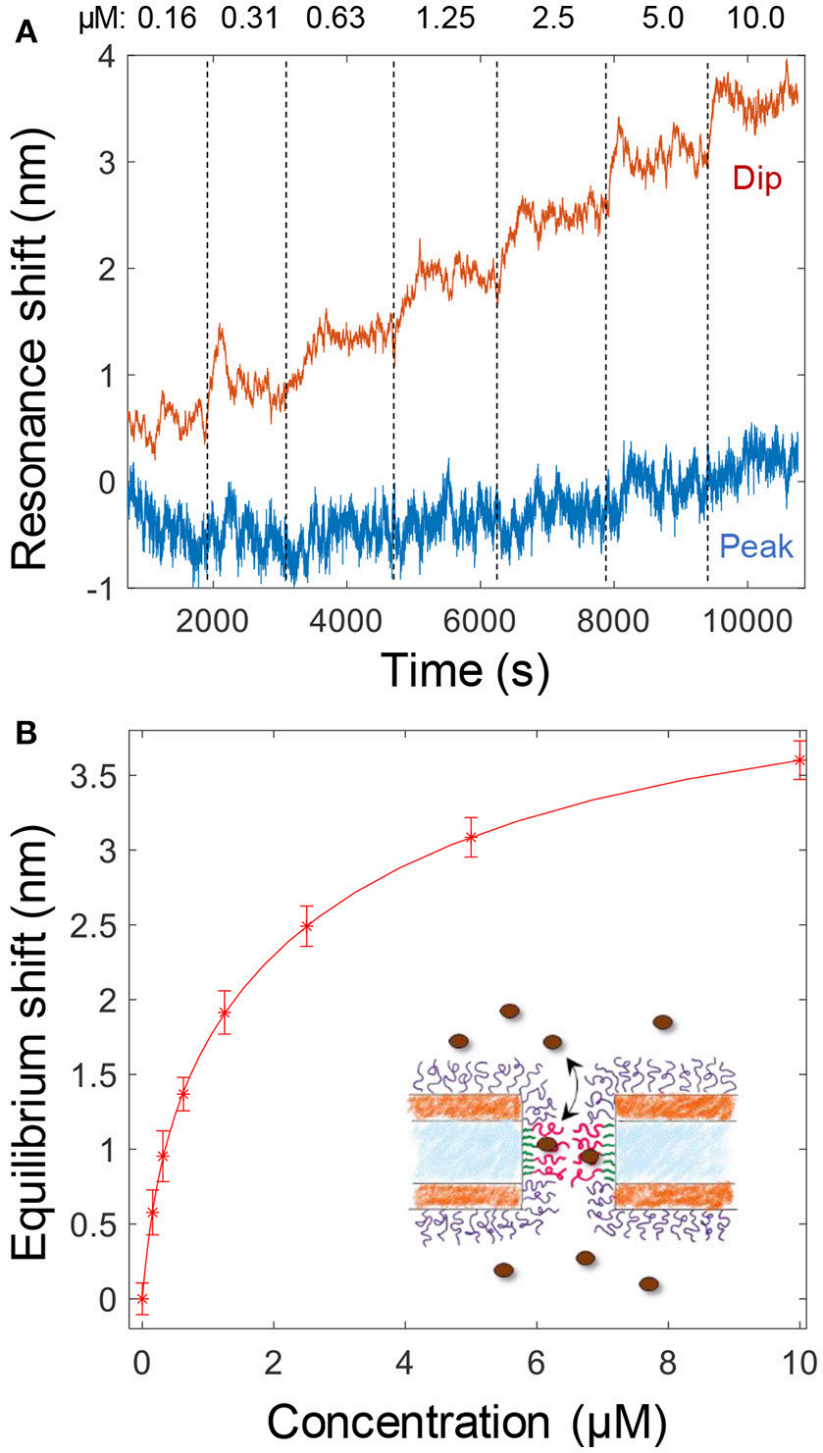
**(A)** Detection and titration of Kapβ1 binding to smaller pores modified with cNsp1. Kapβ1 is introduced at increasing concentrations and the equilibrium signal is obtained by averaging over time (at least 10 min for each concentration, corresponding to ~1,000 values). **(B)** The equilibrium signal for the different concentrations introduced. The curve shows a fit to the two component Langmuir model.

The binding of Kapβ1 plays an important role in NPC barrier and transport function (Kapinos et al., 2017). To mimic the interaction between the transport proteins and the disordered FG domains in the NPC, we added Kapβ1 in increasing concentrations to smaller nanopores functionalized with cNsp1 (Figure 4A). The noise level is orders of magnitude higher because of the very weak spectral resonance (Figure 1D) and it was only for the dip wavelength that signals could be identified in the data. As illustrated in Figure 4A, the peak signal is barely higher than the noise level. Although kinetics could not be monitored due to the high noise, the equilibrium signals could be identified by averaging the dip position over a timescale of ~10 min (Figure 4A). Even if much better signal to noise was obtained by using larger pores (Figure 3), we still consider these data more interesting since the pore diameter is indeed comparable to the inner diameter of the NPC (Akey, 1989; Maimon et al., 2012). Specifically, it would be interesting to study how confinement in a pore geometry might impact the binding interactions of Kapβ1 with the FG domains in comparison to planar SPR surfaces (Schoch et al., 2012; Kapinos et al., 2014). Figure 4B shows the average dip shift after equilibrium establishment at different concentrations of Kapβ1. These values were fitted to a two component Langmuir isotherm (Kapinos et al., 2014):

(1) $$\begin{array}{r} \Gamma \left(C\right)=\Gamma_{1}\frac{C}{C+K_{1}}+\Gamma_{2}\frac{C}{C+K_{2}} \end{array}$$

Here Γ_1_ is the maximum surface coverage of species bound in configuration 1 with affinity constant *K*_1_ and analogously for Γ_2_ and *K*_2_. The configurations represent two different types of binding. This modification of the ordinary single binding site Langmuir equilibrium is necessary to fit data for Kapβ1 interacting with immobilized cNsp1 and originates from the multivalency of the interaction that modulates Kapβ1 binding (Schoch et al., 2012). In brief, transporters may be either loosely or strongly bound depending on the number of interacting binding sites. We fitted *K*_1_ = 341 nM and *K*_2_ = 3.8 μM, which is very close to the values determined on planar surfaces (*K*_1_ = 0.34 ± 0.06 and *K*_2_ = 5.61 ± 1.98 μM) (Wagner et al., 2015). Experiments on larger pores suggested the same affinities. Note also that the data in Figure 4A shows a very high dip signal and negligible peak signal, an effect which is only observed for selective binding inside the pores (Junesch et al., 2015).

Our results indicate that the negative cylindrical curvature inside the nanopore has no strong influence on the binding affinities. However, the main purpose of this work is to illustrate the concept of using plasmonic nanopores as NPC mimics by suitable chemical functionalization. More experiments are needed for extensive quantitative analysis of Kapβ1 binding affinity for different FG domains and pore sizes.

## Conclusions

We have shown APTES and thiol-based chemical functionalization of silicon nitride nanopores with thin gold films on both sides. Label-free plasmonic detection of protein binding inside the pores is possible with high resolution for larger (~150 nm) pores. Smaller pores give much higher noise due to the weak spectral resonance, but it was possible to detect protein binding also inside pores down to 50 nm in diameter. Although we did not observe any differences for Kapβ1 in pores compared to planar surfaces, this still has important implications as it suggests that results obtained from analysis on planar surfaces can be applicable also to the geometry of the NPC. Due to its hourglass shape, the NPC has an inner diameter of ~50 nm at the middle plane and a larger inner diameter of ~70 nm at the nuclear and cytoplasmic rings (Akey, 1989; Maimon et al., 2012). Our small pores have an upper diameter of 80–90 nm and a lower diameter of 50–60 nm and thus are still slightly larger than the interior channel of the NPC. The main limitation with our concept is that the plasmonic nanopores lose their plasmonic activity when the diameter reaches 50 nm. However, a more sophisticated and thicker surface coating on the interior walls would make it possible to reduce the effective diameter while maintaining a large enough pore through the solid materials to keep the plasmonic activity. Since our structures are “true” pores, i.e., they connect two large liquid compartments they form a basis for constructing sophisticated mimics of membranes and barriers between intracellular compartments (Kowalczyk et al., 2011).

## Author Contributions

All authors listed have made a substantial, direct and intellectual contribution to the work, and approved it for publication.

### Conflict of Interest Statement

The authors declare that the research was conducted in the absence of any commercial or financial relationships that could be construed as a potential conflict of interest.
